# Efficacy and Safety of Argon Laser Peripheral Iridoplasty and Systemic Medical Therapy in Asian Patients with Acute Primary Angle Closure: A Meta-Analysis of Randomized Controlled Trials

**DOI:** 10.1155/2019/7697416

**Published:** 2019-05-02

**Authors:** Wenting Cai, Qiyang Lou, Jiaqi Fan, Donghui Yu, Tianyi Shen, Jing Yu

**Affiliations:** ^1^Department of Ophthalmology, Ninghai First Hospital, Ninghai, Zhejiang, China; ^2^Department of Ophthalmology, Shanghai Tenth People's Hospital Affiliated to Tongji University, Shanghai, China; ^3^Department of Ophthalmology, Nanjing Medical University, Nanjing, China

## Abstract

**Purpose:**

The purpose of this meta-analysis was to assess the percent reduction in the intraocular pressure (IOP) after argon laser peripheral iridoplasty (ALPI) and systemic medical therapy in patients with acute primary angle closure (APAC).

**Methods:**

We searched a number of electronic databases, including MEDLINE, EMBASE, PubMed, and Cochrane Library. We searched the electronic databases from the inception of the databases to August 2018. The primary outcomes included the IOP reduction (IOPR), percent reduction in IOP (IOPR%) from baseline to the endpoint and peripheral anterior synechiae (PAS). The secondary outcomes included the cup-to-disc ratio (CDR), mean endothelial count, and percent of patients requiring topical glaucoma medication. Summary weighted mean difference (WMD), odds ratio (OR), and 95% confidence intervals (CIs) were calculated.

**Results:**

Four eligible studies including 183 eyes (92 in the ALPI group and 91 in the medical therapy group) were identified. When comparing ALPI to medical therapy, the WMDs of the IOPR% were 30.03 (95% CI: 21.33 to 38.72, *p* < 0.00001) at 15 minutes, 27.39 (95% CI: 18.89 to 35.89, *p* < 0.00001) at 30 minutes, 18.15 (95% CI: 10.63 to 25.68, *p* < 0.00001) at 1 hour, and 12.91 (95% CI: 4.50 to 21.32, *p*=0.003) at 2 hours. There was no statistically significant difference between the two groups at 24 hours and at more than 6 months after therapy. Meanwhile, no significant difference was observed in the degree of PAS, CDR, mean endothelial count, and percent of patients requiring topical glaucoma medication after treatment between the two groups.

**Conclusions:**

Both ALPI and systemic medications were effective with regard to decreasing the IOP. ALPI was more effective in lowering the IOP within the first two hours. Therefore, ALPI may be a better choice for rapidly lowering the IOP in patients with APAC within a short period.

## 1. Introduction

Acute primary angle closure (APAC) was defined as an eye with an occludable drainage angle, with occurrence of trabecular obstruction by the peripheral iris, resulting in a rapid onset of intraocular pressure (IOP) [1]. Acute primary angle-closure glaucoma (PACG) is a subtype of glaucoma, with the feature of significant glaucomatous damage to the optic nerve and APAC occurrence [2]. PACG and APAC are characterized by shorter axial length, shallower anterior chamber depth, and thicker lenses [3–5]. The patients can present with severe pain, photophobia, tearing, and blurry vision that may be accompanied by halos around lights [6].

Argon laser peripheral iridoplasty (ALPI) has shown to dramatically lower the IOP and open up the closed chamber angles. ALPI applies contractive burns to the peripheral iris, which contracts the peripheral iris stroma and creates a space between the anterior iris surface and the trabecular meshwork. The IOP can be reduced quickly, while the anterior chamber angle reopens [7–9]. ALPI is useful for reversing an attack of an acute angle closure, either as an initial measure or when medical therapies fail [10]. Some studies have found that ALPI is a safe and effective procedure with a satisfactory long-term success rate [11, 12]. Other treatments such as laser iridotomy do not show ideal IOP-lowering effect in Asian patients with APAC. So ALPI is taken into consideration in this study.

Systemic medical therapies include intravenous carbonic anhydrase inhibitors, hyperosmotic agents, and steroids. Carbonic anhydrase inhibitors such as acetazolamide can inhibit ciliary process enzymes and reduce aqueous humor secretion to achieve IOP reduction [13]. Hyperosmotic agents like mannitol had a good effect on lowering IOP. However, systemic medical therapies are associated with some systemic risks, including electrolyte imbalances and congestive heart failure in susceptible individuals [14, 15]. And, some rare but life-threatening complications were also reported previously [16, 17].

At present, several published clinical trials have compared the efficacy of ALPI with systemic medical therapy [18–21]. It remains unclear which treatment option should be recommended as the first-line treatment. The purpose of this meta-analysis was to systematically evaluate the efficacy of ALPI compared with that of medical therapy for the treatment of APAC.

## 2. Methods

### 2.1. Search Strategy

We searched a number of electronic databases, including MEDLINE, EMBASE, PubMed, and Cochrane Library, from inception of the databases to August 2018 with language restrictions. Key terms used for the systematic search were “iridoplasty,” “medical therapy,” “medication,” “primary angle-closure glaucoma,” “acute primary angle closure.” We manually searched the reference lists of the original studies and review articles that were identified with the electronic search for other potentially eligible articles.

### 2.2. Inclusion and Exclusion Criteria

All selected publications were screened according to predefined selection criteria. Eligible studies met the following criteria: (1) study design, randomized controlled trials; (2) population, APAC or PACG without iridotomy performed previously; (3) intervention, ALPI versus systemic medical therapy; and (4) outcome variables, the IOP reduction (IOPR), percent reduction in IOP (IOPR%), peripheral anterior synechiae (PAS), cup-to-disc ratio (CDR), mean endothelial count, and percent of patients requiring topical glaucoma medication. Meetings, abstracts, studies without complete data or with inconsistent or erroneous data, duplicate publications, letters, and reviews were excluded.

### 2.3. Data Extraction and Quality Assessment

Two independent reviewers (Cai WT and Lou QY) examined the electronic searches and obtained the full reports of all citations that were likely to meet the selection criteria. Disagreements were resolved by consensus after discussion. If there were multiple reports for a particular study, data from the most recent publication were extracted.

The following information was extracted: the name of the first author, the publication year, the trial location, the study design, disease, intervention, study population characteristics (age, sex, and eyes in study), the duration of attack, and follow-up durations. A second reviewer double-checked all data. Quality assessments were conducted using the modified Jadad assessment tool [22]. Two review authors independently assessed the risk of bias for each trial, and disagreements were resolved through discussion.

### 2.4. Outcome Measures

We calculated the IOPR% to assess the efficacy of ALPI and medical therapy because the baseline IOP between the two groups was heterogeneous. In brief, if the mean and standard deviation (SD) of the IOPR% were reported, they were used directly. If these data were not available, they were calculated according to the methods described below: IOPR = IOP_baseline_ − IOP_endpoint_ and SD_IOPR_ = (SD_baseline_^2^ + SD_endpoint_^2^ − SD_baseline_ ∗ SD_endpoint_)^1/2^; then the IOPR% and SD of the IOPR% (SD_IOPR_%) were estimated by IOPR% = IOPR/IOP_baseline_ and SD_IOPR%_ = SD_IOPR_/IOP_baseline_ [23, 24].

### 2.5. Statistical Analysis

This meta-analysis was conducted using RevMan5.3 software. Heterogeneity was assessed by calculating the *I*^2^ statistic and by performing a chi-squared test (assessing the *p*-value). An *I*^2^ > 50% was considered to be indicative of significant heterogeneity. Random-effects or fixed-effects models were applied according to the between-study heterogeneity. Summary weighted mean difference (WMD), odds ratio (OR), and 95% confidence intervals (CIs) were calculated. The overall effect was determined to be statistically significant when *p* < 0.05 [25]. The forest plot was generated to show the comparisons clearly.

## 3. Results

### 3.1. Literature Search

**Figure 1:**
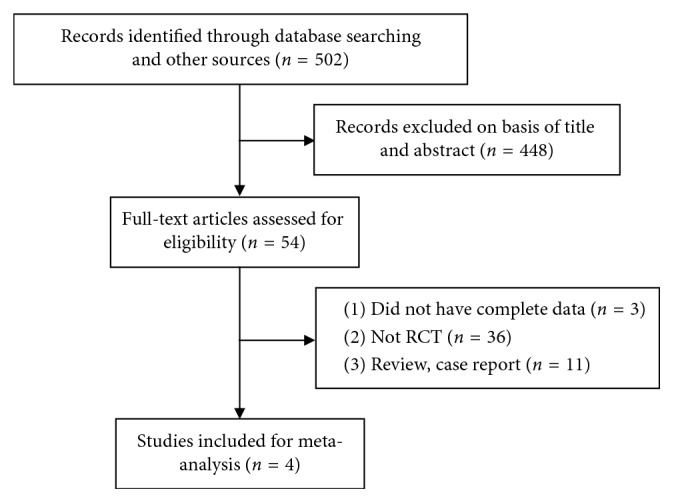
Flow diagram of the literature search in this meta-analysis.

The flow chart for the selection of the articles is shown in Figure 1. The initial search identified 502 studies in English. We excluded 448 studies based on the titles and abstracts. During the examination of the full-text articles, 50 reports were excluded. Finally, 4 RCTs were included in this meta-analysis [18–21].

### 3.2. Characteristics of the Included Studies

The characteristics of the four included RCTs are outlined in Table 1. A total of 183 eyes were evaluated, with 92 in the ALPI group and 91 in the medical therapy group. Three studies were performed in China, and one was performed in Singapore. The duration of attack ranged from 21.6 to 67.2 hours. The duration of follow-up ranged from 1 hour to more than 6 months. In these studies, the patients were randomized into two groups; however, because of the different treatments, the trials did not achieve double-blindness.

### 3.3. Primary Outcomes

#### 3.3.1. IOPR

**Figure 2:**
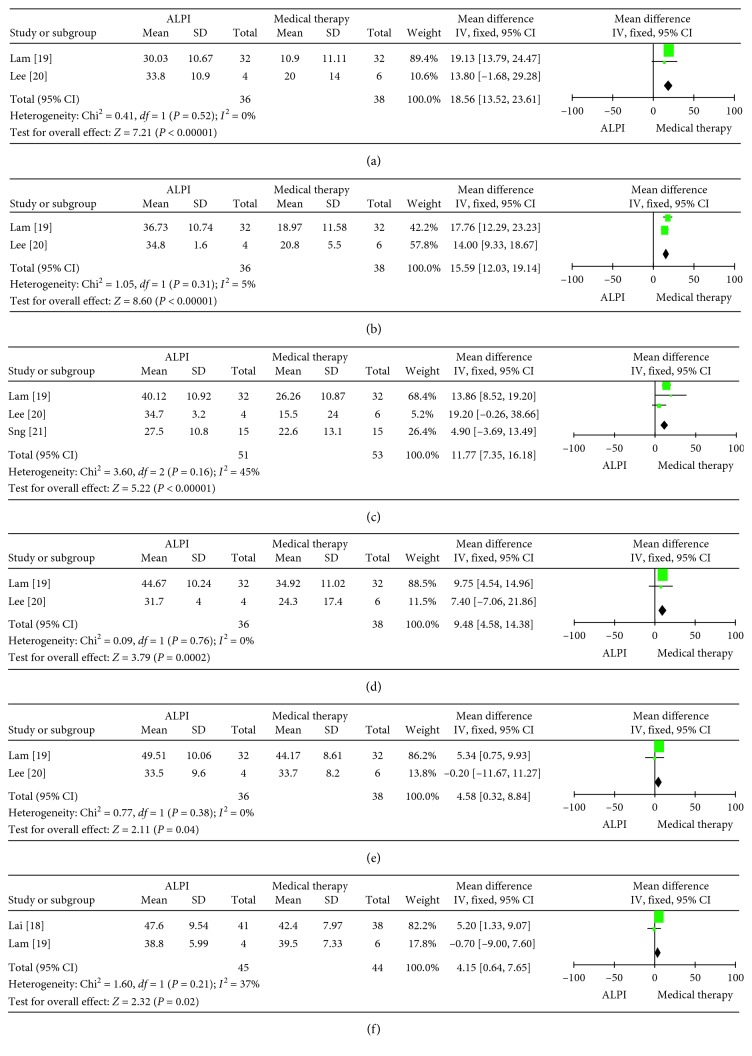
Forest plot comparison of IOPR after treatment with ALPI and medical therapy. (a) 15 min after treatment; (b) 30 min after treatment; (c) 1 h after treatment; (d) 2 h after treatment; (e) 24 h after treatment; (f) >6 m after treatment. IOPR: intraocular pressure reduction; ALPI: argon laser peripheral iridoplasty.

The WMDs of the IOPR in the ALPI group compared with the medical therapy group were 18.56 (95% Cl: 13.52 to 23.61; *p* < 0.00001) at 15 minutes, 15.59 (95% Cl: 12.03 to 19.14; *p* < 0.00001) at 30 minutes, 11.77 (95% Cl: 7.35 to 16.18; *p* < 0.00001) at 1 hour, 9.48 (95% Cl: 4.58 to 14.38; *p*=0.0002) at 2 hours, 4.58 (95% Cl: 0.32 to 8.84; *p*=0.04) at 24 hours, and 4.15 (95% Cl: 0.64 to 7.65; *p*=0.02) at more than 6 months (Figure 2).

#### 3.3.2. IOPR%

**Figure 3:**
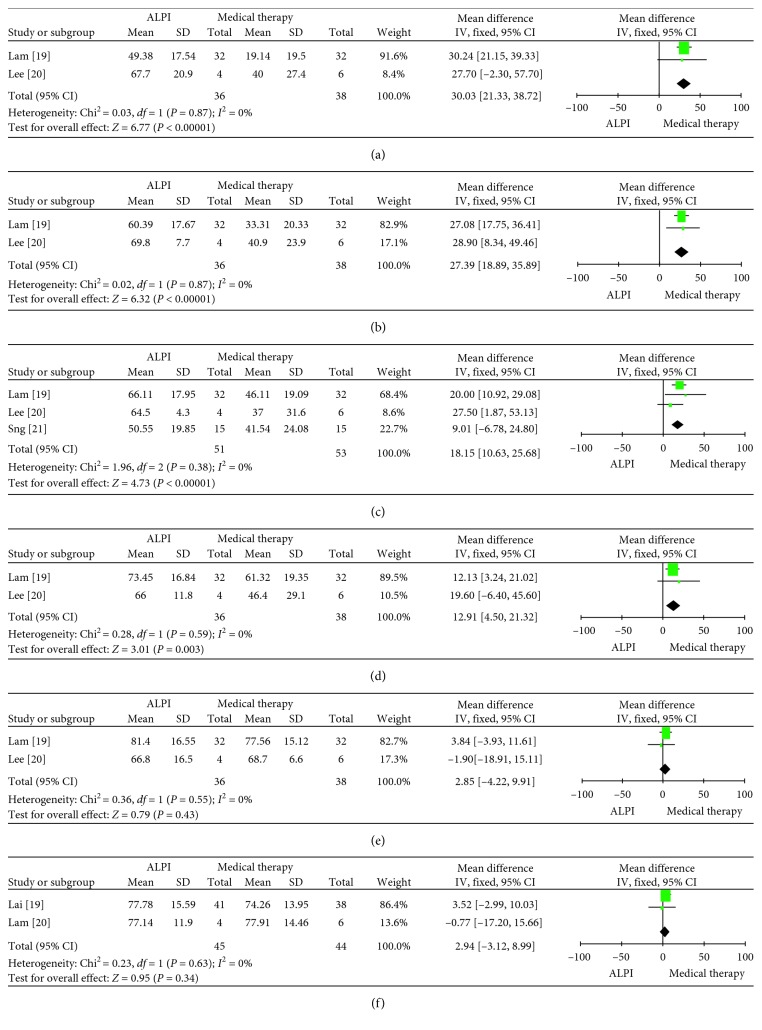
Forest plot comparison of IOPR% after treatment with ALPI and medical therapy. (a) 15 min after treatment; (b) 30 min after treatment; (c) 1 h after treatment; (d) 2 h after treatment; (e) 24 h after treatment; (f) >6 m after treatment. IOPR: percentage reduction in intraocular pressure; ALPI: argon laser peripheral iridoplasty.

Four studies were included in this meta-analysis. The IOPR% in the two groups is shown in Figure 3. The IOPR% in the ALPI group was higher than that in the medical therapy group at 15 minutes (WMD: 30.03, 95% CI: 21.33 to 38.72, *p* < 0.00001), 30 minutes (WMD: 27.39, 95% CI: 18.89 to 35.89, *p* < 0.00001), 1 hour (WMD: 18.15, 95% CI: 10.63 to 25.68, *p* < 0.00001), 2 hours (WMD: 12.91, 95% CI: 4.50 to 21.32, *p*=0.003), 24 hours (WMD: 2.85, 95% CI: −4.22 to 9.91, *p*=0.43), and more than 6 months (WMD: 2.94, 95% CI: −3.12 to 8.99, *p*=0.34).

#### 3.3.3. PAS

**Figure 4:**
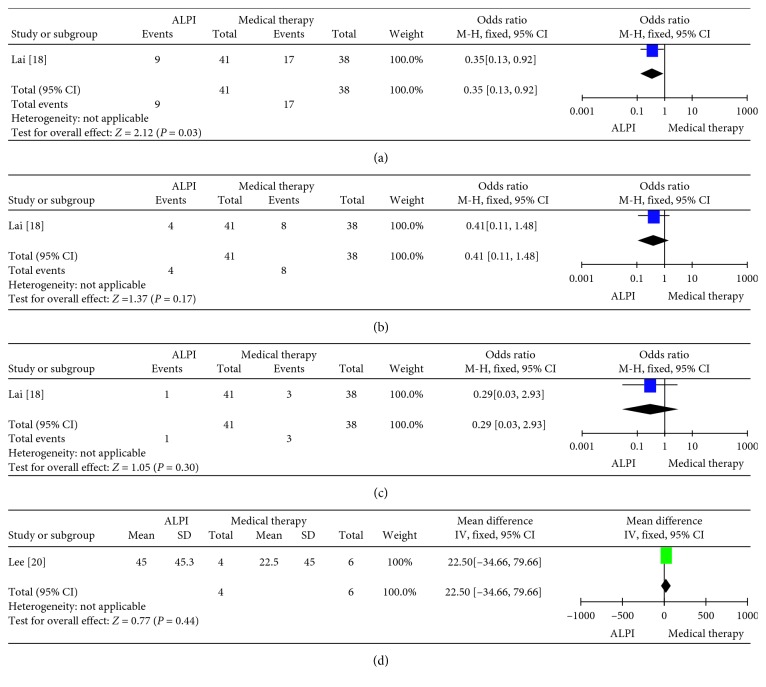
Forest plot showing the numbers of PAS ≥ 90°, 180°, 270°, and the degree of PAS after treatment with ALPI and medical therapy. (a) PAS ≥ 90°; (b) PAS ≥ 180°; (c) PAS ≥ 270°; (d) degree of PAS. PAS: peripheral anterior synechiae; ALPI: argon laser peripheral iridoplasty.

Two trials reported the PAS between these two groups. One study showed the numbers of PAS ≥ 90° in ALPI group was less than those in medical groups (*p*=0.03). There was no obvious difference in regard to the numbers of PAS ≥ 180° and PAS ≥ 180°. The other study showed no difference in the degree of PAS at 3 months (Figure 4).

### 3.4. Secondary Outcomes

#### 3.4.1. Mean Endothelial Count

**Figure 5:**
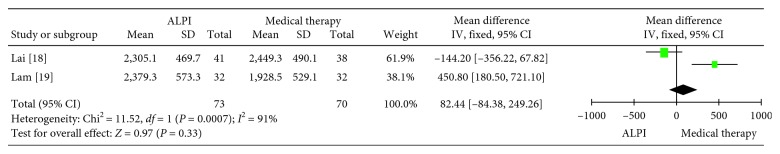
Forest plot showing the endothelial cell count. ALPI: argon laser peripheral iridoplasty.

Two studies were included in this meta-analysis. As shown in Figure 5, the results showed that the mean endothelial count was not significantly different between the ALPI and medical therapy groups (WMD: 82.44, 95% CI: −84.38 to 249.26, *p*=0.33).

#### 3.4.2. Cup-to-Disc Ratio

**Figure 6:**
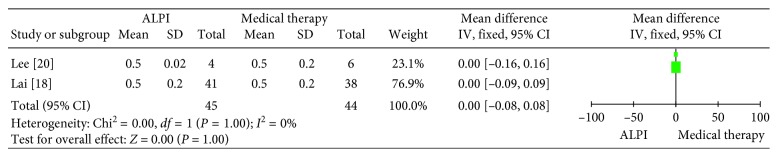
Forest plot comparison of CDR after treatment with ALPI and medical therapy. CDR: cup-to-disc ratio; ALPI: argon laser peripheral iridoplasty.

Two trials reported the cup-to-disc ratio for these two groups. There was no obvious difference between ALPI treatment and medical therapy in regard to the cup-to-disk ratio (WMD: 0.00, 95% CI: −0.08 to 0.08, *p*=1.00). (Figure 6).

#### 3.4.3. Percent of Patients Requiring Topical Glaucoma Medication

**Figure 7:**
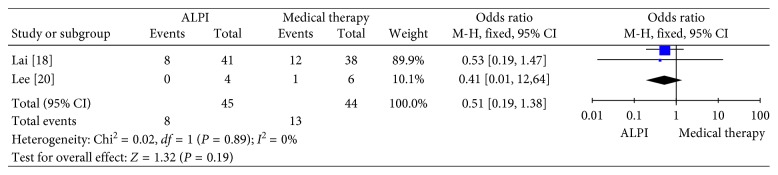
Forest plot showing the percentage requiring topical glaucoma medication. ALPI: argon laser peripheral iridoplasty.

Two trials were included in our further analysis. As shown in Figure 7, there was no obvious difference in the percentage of patients using topical glaucoma medication after ALPI and medical therapy (OR: 0.51, 95% CI: 0.19 to 1.38, *p*=0.19).

## 4. Discussion

Lowering the IOP is the goal of treatment to prevent progressive and irreversible optic neuropathy [26]. It is not recommended to perform surgery like trabeculectomy during a period of acute attack. Both ALPI and medical therapy aim to decrease the IOP rapidly, which was prior to the further treatment [27]. The pooled results from the meta-analysis of four RCTs demonstrate that ALPI reduces the IOP significantly more rapidly than systemic medications in the first 24 hours.

A preliminary study found that immediate ALPI can be used safely and effectively in the first-line management to lower IOP [28]. Vitor et al. suggested that ALPI was a useful procedure independent of the underlying mechanism, leading to angle widening and a moderate IOP reduction in patients with occludable angles [29]. The results showed the rapidity and strength of ALPI in the first 2 hours after treatment. At 24 hours, ALPI was superior to treatment with medications, but the difference was not significant. More than 6 months after treatment, there was no obvious difference between these two groups. However, the results at more than 6 months were not reliable due to the small sample size.

PAS refers to the adhesion of the peripheral iris to the drainage angle, which may be caused by the repeated attack of the appositional closure of the angle [30]. The formation of PAS makes it difficult for the aqueous humor to flow through the chamber angles. Therefore, decreasing the formation of PAS plays an important role in lowering the IOP. Two trials recorded the PAS between these two groups. One study demonstrated that the numbers of PAS ≥ 90° in ALPI group was less than those in medical groups. Since the sample size and included trials were small, the difference of PAS between two groups should be verified with further randomized trials.

In addition, no significant difference was observed in the CDR, mean endothelial count, and percent of patients requiring topical glaucoma medication after treatment between the two groups. ALPI may be related with potential risks such as corneal burn by laser corneal endothelial cell loss. However, mean endothelial count showed no significant difference between ALPI and medical therapy, which indicated that ALPI is safe and have no damage on cornea to some extent. In addition, ALPI may also be associated with iris atrophy, but the incidence is rare.

There are some limitations in our meta-analysis that should be taken into consideration. First, we eventually included 4 RCTs and 183 eyes in this meta-analysis. The studies were carried out with small sample sizes, and the descriptions of the performance bias, detection bias, and reporting bias are not clear, which may affect the reliability of the results. Second, all participants in the studies were Asian; thus, these results may not be generalizable to other races, such as European individuals. Third, only two studies had a follow-up duration of more than 6 months, so more RCTs with long-term follow-up are necessary in the future.

Considering all the above information, this meta-analysis should be regarded as an indicator that ALPI is effective in treating patients with APAC during the first two hours. The side effects of this treatment still need to be considered, and further study is required to demonstrate the long-term follow-up between ALPI and systemic medications. This meta-analysis is intended to serve as evidence for the use of ALPI in clinical treatment.

In a nutshell, the present meta-analysis showed that both ALPI and systemic medications are effective with regard to decreasing IOP. ALPI is more effective in lowering the IOP within the first two hours. Therefore, ALPI may be a better choice to rapidly lower the IOP in patients with APAC.

## Figures and Tables

**Table 1:** Characteristics of the six included randomized controlled trial.

Studies	Year	Location	Design	Sex (M/F)	Intervention	Eye	Age (mean ± SD)	Duration of attack (h)	Follow-up	Jadad score
Lam	2002	China	RCT	51/13	ALPI	32	68.38 ± 10.39	34.66 ± 43.06 h	24 h	3
					Medical therapy	32	68.06 ± 9.16	31.11 ± 31.77 h		
Lee	2013	China	RCT	3/7	ALPI	4	78.3 ± 11.0	0.9 ± 0.25 d	≥6 m	3
					Medical therapy	6	79.7 ± 7.2	2.8 ± 3.5 d		
Lai	2006	China	RCT	61/10	ALPI	41	70.0 ± 10.5	41.6 ± 47.6	≥6 m	3
					Medical therapy	38	66.5 ± 8.5	29.7 ± 23.8		
Sng	2015	Singapore	RCT	13/17	ALPI	15	61.9 ± 9.0	NA	1 h	3
					Medical therapy	15	63.7 ± 6.3			
